# Testing of the assisting software for radiologists analysing head CT images: lessons learned

**DOI:** 10.1186/s12880-017-0229-1

**Published:** 2017-12-11

**Authors:** Petr Martynov, Nikolai Mitropolskii, Katri Kukkola, Monika Gretsch, Vesa-Matti Koivisto, Ilkka Lindgren, Jani Saunavaara, Jarmo Reponen, Anssi Mäkynen

**Affiliations:** 1 Optoelectronics and Measurement Techniques Unit, University of Oulu, PO Box 4500, 90014 Oulu, Finland; 2 Department of Radiology, Oulu University Hospital, PO Box 50, 90029 Oulu, Finland; 3 Department of Radiology, Lapland Hospital District, PO Box 8041, 96101 Rovaniemi, Finland; 4 Medical Imaging Centre of Southwest Finland, Turku University Hospital, PO Box 52, 20521 Turku, Finland; 5 Finntelemedicum, Research Unit of Medical Imaging, Physics and Technology, University of Oulu; Department of Radiology, Hospital of Raahe, PL 25, 92101 Raahe, Finland

**Keywords:** Computer software, Search engine, Brain imaging, Computed tomography, Research design

## Abstract

**Background:**

Assessing a plan for user testing and evaluation of the assisting software developed for radiologists.

**Methods:**

Test plan was assessed in experimental testing, where users performed reporting on head computed tomography studies with the aid of the software developed. The user testing included usability tests, questionnaires, and interviews. In addition, search relevance was assessed on the basis of user opinions.

**Results:**

The testing demonstrated weaknesses in the initial plan and enabled improvements. Results showed that the software has acceptable usability level but some minor fixes are needed before larger-scale pilot testing. The research also proved that it is possible even for radiologists with under a year’s experience to perform reporting of non-obvious cases when assisted by the software developed. Due to the small number of test users, it was impossible to assess effects on diagnosis quality.

**Conclusions:**

The results of the tests performed showed that the test plan designed is useful, and answers to the key research questions should be forthcoming after testing with more radiologists. The preliminary testing revealed opportunities to improve test plan and flow, thereby illustrating that arranging preliminary test sessions prior to any complex scenarios is beneficial.

## Background

Content-based image retrieval (CBIR) in radiology grew to a popular research topic in recent years [1] aimed to make workflow of radiologists more effective in case of increasing numbers of patients and medical images worldwide. The general purpose of CBIR is to help users to find similar items in some set of images, which is especially applicable for automation in radiology. Since the entire field of CBIR technology still faces challenges, evaluation of the usefulness of the CBIR solution applied is also challenging. There are reports addressing implementation and evaluation of applications designed to aid in analysing computed tomography (CT) images [2, 3], mammograms [4, 5], x-ray images [6–8], and other modalities or complex solutions [9–11]; however, though all those systems are based on CBIR technology, they differ greatly in their workflow and implementation. Therefore, researchers have developed original testing scenarios specific to the case at hand. Such scenarios have typically taken only one aspect of the system into account. The same issue has manifested itself also in other biomedical applications of CBIR [12, 13]. The approach in earlier research has either concentrated on validation of the retrieval system’s performance and result quality with users [2, 3, 5, 6, 12] or on usability issues of the solution developed [8, 9, 11, 13]. Notwithstanding significant amount of experience and knowledge that has been accumulated regarding to evaluation of CBIR systems, there is still no comprehensive, unified approach to testing scenarios. This lack is especially evident if one plans to deal with complex scenarios or test a novel solution that influences radiologists’ existing workflow.

In the CARDS (Computer Assisted Radiology Diagnosis System) project [14], the software application named SeeMIK was developed. The main purpose behind the application was to assist in radiologists’ interpretation of CT images of the head by providing tools for image and/or text-based search in hospital’s Picture Archiving and Communication System (PACS) and Radiological Information System (RIS) databases. It was designed not to perform diagnosis but as an extension to RIS and PACS for retrieving meaningful textual and image data from them.

In consideration of experiences outlined in the papers mentioned above, a scenario for multifaceted evaluation of the software was designed. The general idea was to find answers to the following questions:

- Are the search results relevant?
- Does the software provide the required level of usability?
- Are the search results useful in decision-making?

The target was to cover the software usability and quality of the search results with a single test process. Due to the complexity of the process, it was decided that preliminary testing of the software should be performed, to verify whether the testing plan was designed well and one could obtain meaningful results and minimise mistakes and systematic bias.

## Methods

Two junior radiologists, with six and twelve months of experience, were recruited from Raahe Hospital, Finland, for preliminary testing. Both of the participating radiologists had a separate workroom and computer workstation, and their work was followed by observers during all testing sessions.

### The software under test

As a conventional CBIR system the SeeMIK software implements two processing modes: indexing and search. Indexing is needed to determine features (descriptors) for every image in the dataset according to selected algorithm. Features are quantitative characteristics (i.e. color data, textures, shape, size, and location of objects) of visual content, which are necessary for fast image comparison and retrieval. In search mode, conventional CBIR system assesses similarity between image features passed as a query and features stored in the index. Result images returned to the user in a decreasing similarity order.

In the software developed indexing is carried out in two phases: initial indexing (after installation and before normal functioning) and continuous background indexing. In the first phase, a sufficiently large corpus of head CT studies (numbering in the thousands) and associated reports from the hospital’s PACS and RIS are used to build a ‘bag of visual words’ (BoVW) [15] vocabulary for the images and a textual vocabulary. All words in reports are stemmed beforehand so they are indexed in a truncated form. The software does not recognize which terms are significant for diagnosis and consider all the words have the same weight. Then, the software creates an initial ‘inverted index’ [16], composed of stemmed words and ‘visual words’, extracted from texts and images of the studies, respectively. In the second phase, it continuously obtains not-yet-indexed studies from PACS and RIS and indexes them using the existing vocabularies.

**Fig. 1:**
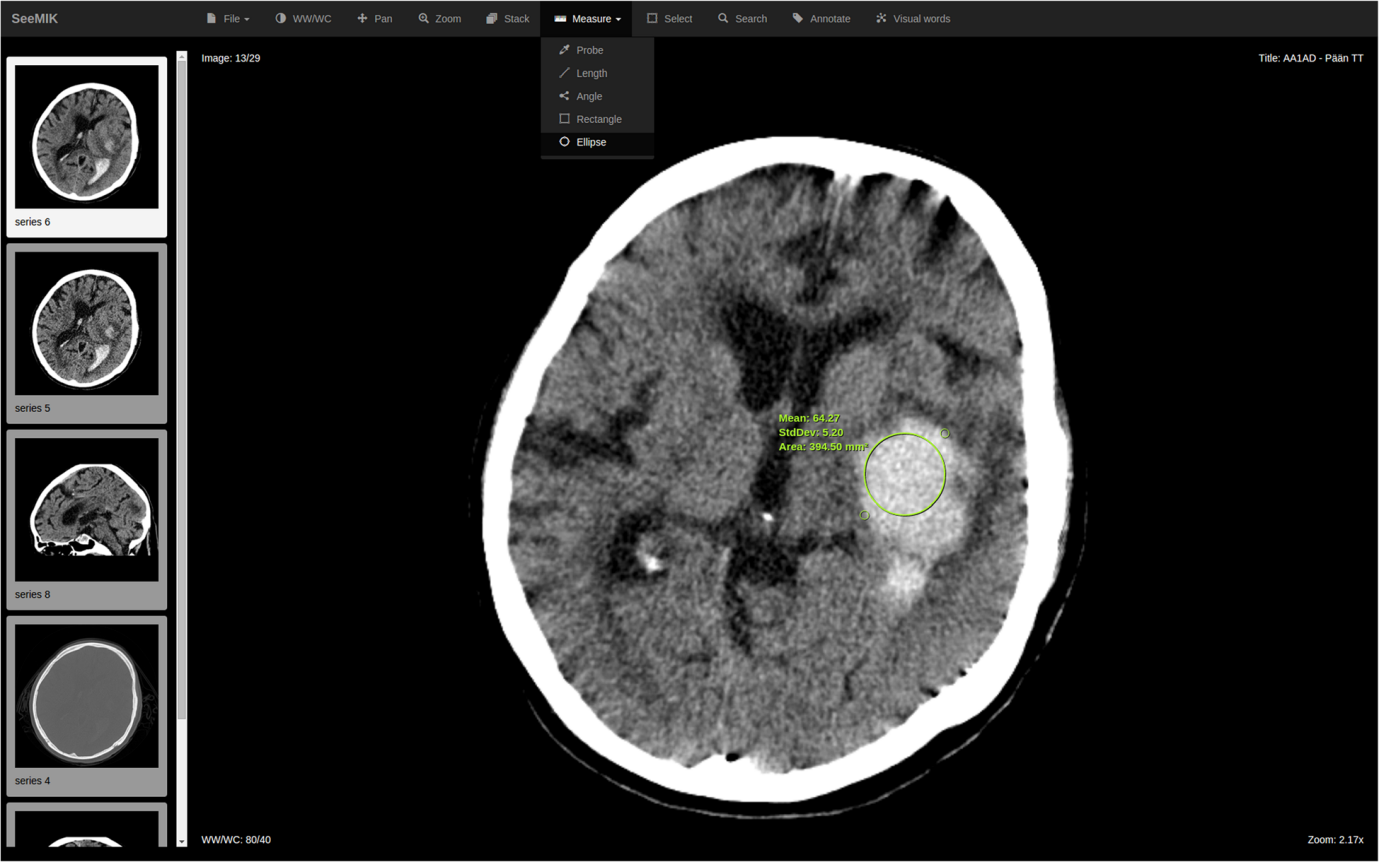
The user interface of the DICOM viewer

**Fig. 2:**
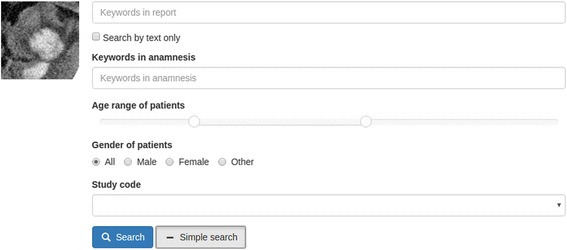
Expanded search form with the selected area of interest as a query element

In search mode, the user interface of the software resembles a typical Web-based DICOM (Digital Imaging and Communications in Medicine) viewer connected to the PACS, which allows radiologists, for example, to browse and manipulate study images, measure tissue density (see Fig. 1). Unlike a conventional viewer, one can also search for similar images or studies (see Fig. 2) and view the search results found from the PACS and RIS of the hospital.

Text, an image, a selected part of an image, or a combination of image and text can be used as the search query. For textual queries, the software determines stemmed forms of words of the provided phrase and then looks for their combinations in the index. For image queries, the search engine developed processes the input image and compares its features, described by the existing vocabulary, with image features in the index database. Those images sharing enough ‘visual words’ in common with the query image are returned as search results. For combined queries, the search engine shows results matching both text and image on top and those are followed by results matching either image or text queries independently. In addition, it has so called advanced-search mode, which enables limiting search results by additional parameters such as patients’ age range, patients’ gender and radiological study codes.

**Fig. 3:**
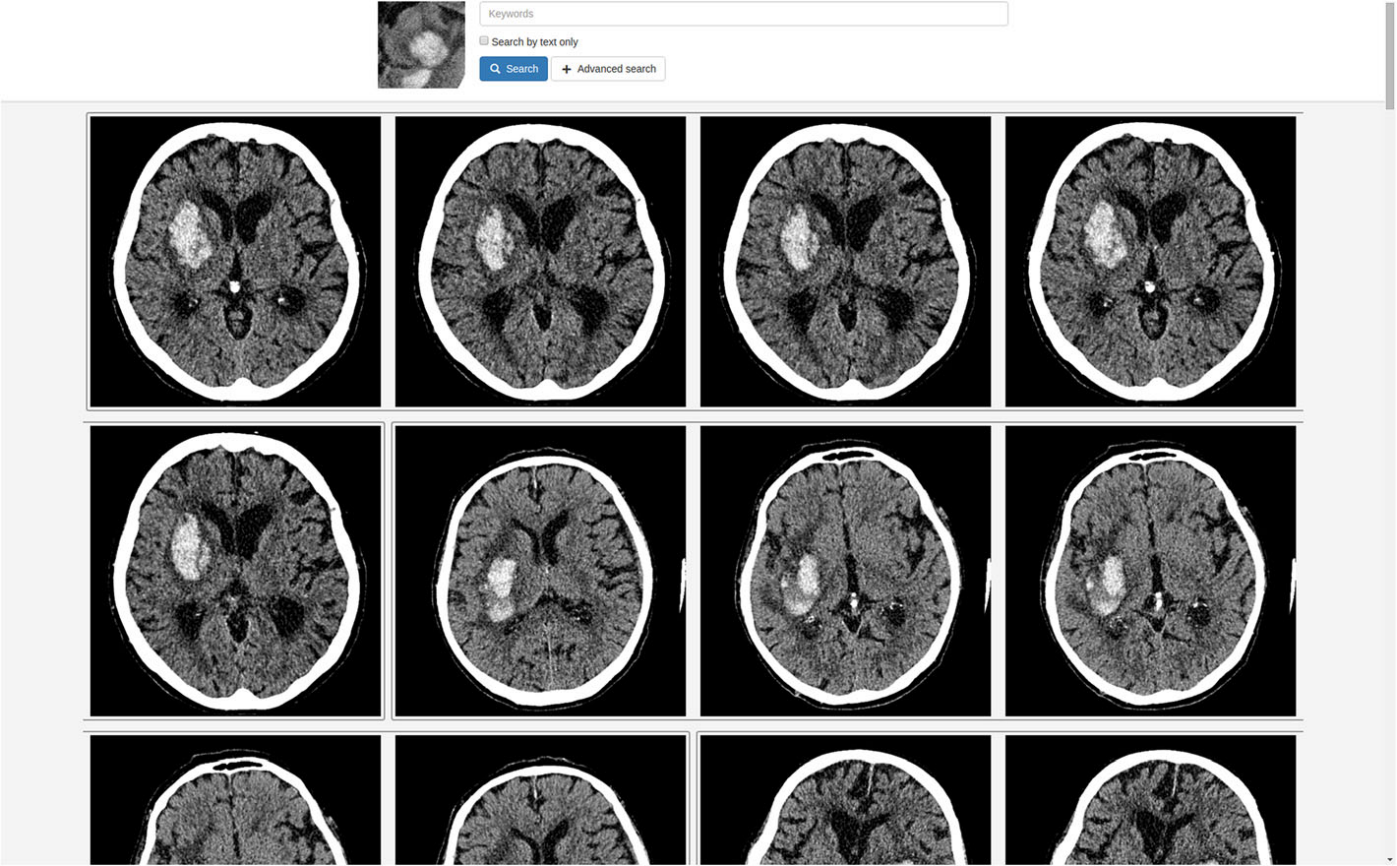
Concise search form and presentation of image-search results in the software

The software presents the results as a list of studies/images matching the query. They are shown as blocks, in decreasing-relevance order, and there can be up to 100 radiological reports (studies) for a text-based search and images, grouped by study, for an image search (see Fig. 3). The search engine finds and shows up to five images for each study on results page. The user can open study images for every search result in the DICOM viewer.

**Fig. 4:**
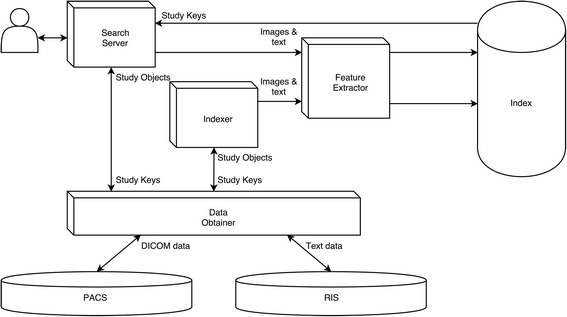
Functional diagram of the software developed. Search server is a user interface module with which users perform searches and view radiological images. Feature extractor is a central module implementing feature extraction, vocabulary creation and text processing algorithms. Index is a file storage containing extracted image features, text terms and created vocabularies. Indexer is a module for performing initial and/or background processing of available image and textual data. Data obtainer is an integration interface for communicating with PACS and RIS

During the testing, the software ran on a server in an isolated network created for the CARDS project at the University of Oulu premises. Architecture of the system developed represented by several connected modules with separate functions (see Fig. 4).

### The testing materials

The Regional Ethics Committee of the Northern Ostrobothnia Hospital District approved this study design and the Northern Ostrobothnia Hospital District gave permission for the registry based data gathering and use for the software development and testing. Only cases which occurrence were high enough were exported to prevent identification after personal data removal. Sample data was exported from the emergency radiology department of Northern Ostrobothnia Hospital District. Radiological studies collected represented all emergency cases processed in the hospital for the last few years. Data exported contained conventional CT studies, enhanced CT studies and mixed studies with both types of CT images made on several different scanners. Text information exported consisted of radiological reports and anamneses linked to image data. For the preliminary testing, a sample of 5975 head CT studies (comprising more than 3.5 million radiological images) was indexed and used. All radiological studies for the test tasks were selected and prepared by an experienced radiologist specializing in neuroradiology, one of the authors of this paper. In total, 16 medical terms and 16 studies, with a wide range of findings, were collected for the relevance testing for the search engine. A study with an obvious finding (intracranial haemorrhage) was chosen for usability testing. For the reporting session, 10 head CT studies were selected in accordance with the following rules:

- The study should not have a conclusion that is obvious to inexperienced radiologists; that is, in a normal work situation, the radiologist would refer to some additional means (literature, an Internet search, or consultation of colleague, for example) to support the decision on the report.
- The CT study should be an appropriate one for observation and decision-making.

Opinions and perceptions of users were collected in several ways: via observation, questionnaires, and interviews. The System Usability Scale (SUS) [17] questionnaire was used to collect first impressions after the radiologists had used the search function, and the Usefulness, Satisfaction, and Ease of use (USE) [18] questionnaire was utilised after the software had been used in a situation mimicking normal work. Interviews were conducted immediately after filling in of the forms, so as to collect richer information on users’ ideas and perceptions of the test and the software. A structured framework was used for the interview questions. All interviews were carried out in the Finnish language, and the audio was recorded, for later transcription and translation into English.

The SUS questionnaire is a proven and reliable tool for measuring usability of a wide variety of software products and services [19]. While quite brief, consisting of only 10 statements, each with five response options for respondents – from ‘strongly agree’ to ‘strongly disagree’ – it highlights the user’s general perception of the software. The USE questionnaire contains 30 well-formulated statements in English, each of which can be assessed with a Likert-type scale from 1 to 7 (representing ‘strongly disagree’ and ‘strongly agree’, respectively) or marked as ‘not applicable’ for the current circumstances. It enables assessing four aspects of software usability: usefulness, ease of use, ease of learning, and satisfaction.

### The testing scenario

**Fig. 5:**
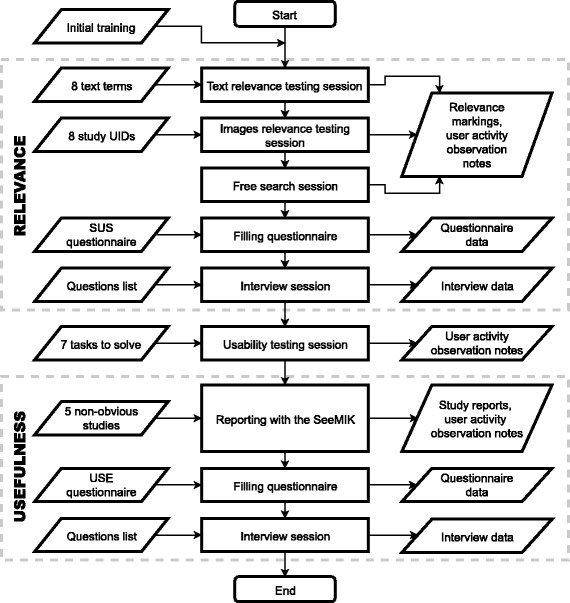
The flow of the preliminary testing carried out

The preliminary testing addressed three factors: relevance of search results, usability, and usefulness (see Fig. 5). Before testing, the participants were instructed and trained to use the software.

The main aim in the **testing of search results’ relevance** was to ascertain a relevance rate for images and studies found by the search engine in the test environment and quantitatively estimate the search results’ quality with the aid of the radiologists. The relevance of the search results was assessed by means of three tasks for the participants:

- Each radiologist was provided with a list of eight medical terms or phrases in Finnish for performing text-based searches. The task for the radiologists was to mark each of the first 10 results for every phrase as a relevant or irrelevant finding with respect to that phrase.
- Each radiologist was provided with a list of eight head CT studies. For each study, the participant’s task was to choose a key image, identify an area of interest, and perform a search by that part of the image. The test materials did not inform the radiologists of the main findings in the associated reports, but it was possible to view the reports in the embedded DICOM viewer. The task for the radiologists was to mark the first 10 images returned as relevant or irrelevant for every search query or, if the first 10 results featured no relevant items, identify at least one relevant image from among the results.
- The final task involved free use of the search functionality by the radiologists. They were able to try searches by both image and text (combined-search) or advanced-search mode. Radiologists were asked to mark at least one relevant finding from the result set returned.

For testing purposes, buttons were added to the user interface for marking the relevance of every image and radiological report in the search results. With these buttons, the radiologists could mark the results as relevant or irrelevant with respect to the search query, and all of their markings were permanently stored in the database of the software. For the first two tasks, the ‘precision at 10’ metric was calculated for each textual or image-based query. The results in the last task were assessed qualitatively. The relevance testing was completed with the SUS questionnaire and interviews with both participants on their first impressions of the software and the test flow.

The main purpose behind the **usability testing** was to ascertain the radiologists’ perceptions of the user interface developed, its suitability for image searching, and its applicability in radiologists’ workflow. The tasks in the associated testing session were derived from possible use cases and eventual needs of radiologists. These included opening a study, checking the anamnesis (study request) and the details of the study, performing a search and opening its results in the viewer, and refining a search via additional criteria. Activity of users on the screen and facial expressions were recorded in the manner typical in such testing. Because of the number of participants, the assessment was qualitative in nature, no quantitative metrics for user interactions (e.g., task-completion time) were collected. Since observers followed all testing sessions, the relevance testing and reporting sessions were targeted also for their ability to reveal usability mistakes and bottlenecks in the user interface.

The primary aim with the **usefulness testing** was to determine the real-world utility of the solution for radiologists who need to report on a non-obvious study. In this connection, the target in the preliminary testing was not to compare the time used for reporting with and without the software but solely to determine whether it is possible to report on the study with SeeMIK alone. In this testing, the junior radiologists were asked to open five specially selected head CT studies in the built-in viewer, analyse them, and report on them. The last two studies in each radiologist’s list were optional. The information provided on those cases was restricted to the DICOM images and anamnesis. Other background details or previous studies for the same patient were unavailable. When reporting on each case, the radiologists were free to use all tools available in the software, without restriction, and dictate the reports created to a voice recorder. The second questionnaire (USE) and the final interview were completed after all other testing was finished, to gather feedback and cover all experience gained by the radiologists in use of the solution developed.

The preliminary testing was performed in two phases and took approximately five hours for each participant. It can be summarised thus:

- In the first phase, the relevance of the search results was estimated and usability issues were identified via the SUS questionnaire, the interview and observation of radiologists performing tasks.
- In the second phase, usability testing with several typical use cases was performed, with follow-up via the usefulness testing session, the USE questionnaire, and the interview.

## Results

The tests showed that it is possible to carry out the desired measurements and collect perceptions of users with the tests used. Although results from actual testing and, especially, questionnaires in preliminary testing with only two participants are, naturally, insufficient for reliable interpretation in the statistical sense, they still yield some insights. Therefore, interviews and observations made during testing are valuable sources of information for evaluation of the successfulness of the test set-up.

Results of relevance tests revealed differences in assumptions as to what was considered relevant between an engineer’s and a radiologist’s perspective. The engineering view of text search was that a study is relevant if the text searched for appears in text related to that study. At the same time, radiologists in the test conditions assumed a text-search result to be relevant only if the report for the study actually contained the search term in its finding or diagnosis section and sometimes they looked for possible confirmation also in the study images. Differences in interpretations of relevance affected the results, and the average ‘precision at 10’ was 81% for text search. Most of the results deemed non-relevant contained sought-for terms in the anamnesis part as a question, but without confirmation of such finding in the report part. Though the software attempted to exclude various forms of negative expressions from the results returned, it did not succeed fully in this, especially because there are multiple ways to express both medical terms and their negatives in the Finnish language.

Image search also showed differences in interpretation of what constituted similarity. The engineers and the search engine judged images to be similar on the basis of visually similar features: areas and edges of areas. For the radiologists, however, the images had to be relevant also in a medical sense, since many different findings can look visually similar (for example, acute haemorrhages and some tumours). In some cases, it was difficult for radiologists to evaluate relevance of images from the results page view alone, so confirmation of similarity was sought in report texts and additional images in the studies found. The software showed up to five images for a study on its results page, so in a few cases the first 10 images represented only two studies in all. The precision at 10 calculated for image search was 39%. Nevertheless, SeeMIK offered at least one relevant study in 93% of image-search attempts.

After the tests, it was noted that one radiologist had accidentally skipped a task in the image relevance test, so only 15 results were gathered in total, rather than the expected 16. When checking the relevance markings collected for analysis after the image-search test, it was noticed that the test users had made a few incorrect markings: indicating relevant images to be irrelevant and vice versa. Occasionally, the image part selected for a search was not the targeted main finding of the study, because the exact finding to search for was not specified in the tasks for the participants.

One of the ideas behind the free-search task was that radiologists could ‘play with’ the search engine and explore it, but it seemed that the participants were very confused and did not know how to start doing so. With a little guidance by the observers, the radiologists tried advanced-search mode and combined-search by using images and text. Both search modes demonstrated good relevance in a few cases tested because the queries were more specific and accurate.

Because the tests began with relevance testing, a few usability issues, such as inadequate visual assistance and unclear behaviour of the user interface controls, were detected at the very outset. All tasks in the usability testing were completed successfully, but during a couple of them, unclear wording in the test guides prompted the radiologists to demand some assistance from the observers. The usability scores from the questionnaires were at a reasonable level: the SUS questionnaire score in both cases was 77.5 out of 100 points, which can be interpreted as showing ‘good’ usability according to ‘SUS: A Retrospective’ [19]. In the interviews, both radiologists raised some minor issues with the user interface and also mentioned a few features they would like to see in the DICOM viewer for reporting, such as multiplanar reconstruction (MPR) and support for several layouts of the working area. However, no serious bugs or stability issues were commented. Both radiologists reported a large number of irrelevant images in some cases, but still they thought the software to have good usability. The search process took time, but neither of the participating radiologists found this to be an issue. As most positive aspects of SeeMIK, the users identified ease of use and that the software is ‘a good tool also for general learning’.

In the usefulness testing, one radiologist reported on four studies, the other on three. The reports they created were checked by an experienced radiologist and compared to the original ones made at the hospital. The reports created with the assisting software were deemed of good quality in their description of the pathology and in their diagnostic assessment. There were some minor errors such as missing a finding or incorrectly interpreting a finding, but these had only minor effect on the quality of the report overall. One study report created was more correct than the original report, but for another study, the original report was more correct than that created during test.

The opinions of radiologists collected via the USE questionnaire and interviews were assessed. Analysis showed generally positive results from the questionnaire: the median was six on the seven-point Likert-type scale for both radiologists. The statements receiving extreme responses were ‘It is useful’ (7), ‘It makes the things I want to accomplish easier to get done’ (7), and ‘I can use it successfully every time’ (3). Both radiologists considered the example cases to suit the testing purposes well: the studies consisted of single findings with a clear place to focus but were still such that junior radiologists without help of the software would have routinely consulted a more experienced radiologist for aid and going through the study together. The software was perceived as useful. The less experienced of the radiologists stated that the solution developed could be a good aid because it offers a reliable, quick, and easy way to compare and verify images. It was described as very interesting for students because information can be searched for in many ways. Especially with unfamiliar findings, it led the participants on the right track. Image search found at least some images for which the findings were suitable, and then it was possible to continue with word search or combined search.

## Discussion

Many aspects of the assisting software were evaluated directly or indirectly during the testing process, and it yielded experience and knowledge that were highly useful for larger-scale testing scenario design. The testing functioned well as a preliminary step, as it revealed weaknesses in the test set-up and the guidance given to the participating radiologists. In addition, it highlighted a few usability issues that should be resolved before larger-scale testing begins. The DICOM viewer used as the user interface had limited functionality in comparison to the software usually used in clinical work, but the participants considered it, for the most part, adequate for the search function.

### The relevance test guidance should be extended

The results of relevance testing revealed that judgement of relevance differed slightly between radiologists and the developers of the software. The initial assumption was that conception of relevance would be intuitive for a radiologist, so it was not described in the testing guides. The test results showed, in contrast, that the concept of relevance should be clearly and precisely defined, lest radiologists employ different interpretations, which could bias results related to search relevance. In addition, for image relevance testing, the testing radiologists should be supplied with the main finding of the study at hand, to make them select the targeted finding.

### The relevance test assessment should be modified

The approach for calculating image-search results relevance should be changed. It became clear that, since the search engine grouped image results by study and showed up to five images related to a single study, marking only the first 10 images may result in incomplete assessment, because in some cases these images represent only two studies. Therefore, it was concluded that, while suitable for studies, calculating the ‘precision at 10’ metric would not be applicable for images in further tests.

### The initial training and testing conditions should be rethought

A need was identified by observing user behaviour and issues such as accidental task-skipping in the preliminary testing: better and longer initial training could leave participants more confident in what is expected from them and, hence, both less nervous and more attentive. At the same time, the accuracy of task performance should be managed better by the observers during the tests, with assistance given as needed.

### Usability testing should be rearranged

The usability testing should be scheduled as the first test, for capturing the first hands-on experience of users. In this case, it may also serve to shape the attitude of participants better, since the tasks are relatively easy and should not impose an excessive cognitive burden. Additionally, the usability testing tasks should be refined such that they are clearer and more natural for the participating radiologists.

### Free-search task should be replaced

The free-search task proved confusing, so it is probably better to use combined-search testing instead. This can be designed as a continuation or further refinement of the image-search task wherein text terms supplement the image-search queries after evaluation of purely image-based results.

### Text-search relevance can be improved via changes in software functioning

The software should search only the report text by default. Thereby, the questions in the anamnesis are excluded from the results and more relevant results are provided. Also, filtering out of negation should be improved by defining and adding new filtering rules.

## Conclusions

**Fig. 6:**
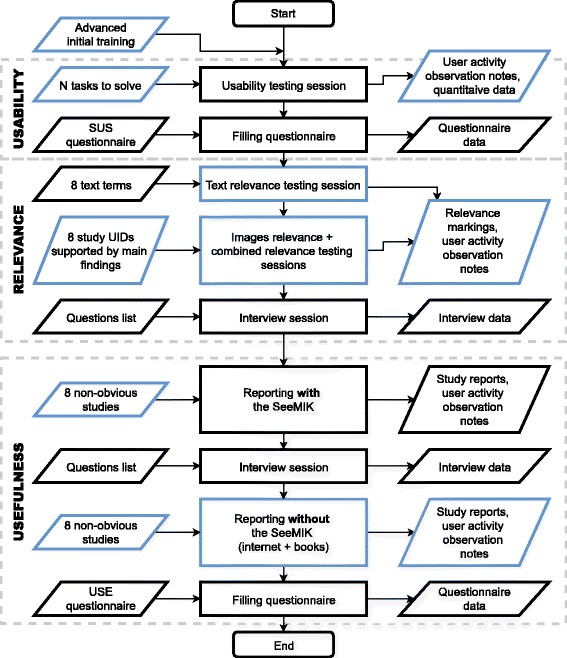
The improved flow for pilot testing, with the changed steps highlighted

Finally, the results of the tests performed show that the test plan designed is useful and that it should be possible to answer the main research questions after testing of the software with more radiologists. Useful qualitative data and opinions of prospective users were collected. Moreover, the assumption was validated that even junior radiologists can report on non-obvious cases with the aid of the assisting software. The tests performed enabled improvements to the test conditions and materials (see Fig. 6) and preparation of a more mature version of SeeMIK, for testing with a larger user group. It seems clear that when one’s test plan cannot be evaluated with all planned conditions (a large enough group of users, the final test materials, sufficient data samples, etc.), simplifying or scaling down the original scenario and performing a test on its basis is still worthwhile. Moreover, with such tests it is possible to provide motivation for further development and gather new ideas and proposals from real users.

## Abbreviations

BoVW: Bag of visual words
CBIR: Content-based image retrieval
CT: Computed tomography
DICOM: Digital imaging and communications in medicine
MPR: Multiplanar reconstruction
PACS: Picture archiving and communication system
RIS: Radiological information system
SUS: System usability scale (questionnaire).
USE: Usefulness, satisfaction, and ease of use (questionnaire).
